# First-Principles Study of Strain Engineering Regulation of SnSe Thermoelectric Properties

**DOI:** 10.3390/ma18174219

**Published:** 2025-09-08

**Authors:** Haoru Zhang, Songqing Zhao, Yuhong Xia, Xinyue Zhang, Lulu Zhou, Zhenqing Yang

**Affiliations:** 1 College of Science, China University of Petroleum, Beijing 102249, China; 2 China University of Petroleum-Beijing at Karamay, Karamay 834000, China

**Keywords:** thermoelectric materials, strain, thermoelectric properties, SnSe, first principles

## Abstract

To study the effect of strain engineering on the thermoelectric properties of SnSe, we combined first-principles calculation and Boltzmann transport theory to study the effect of −4% to 4% strain on SnSe thermoelectric properties. Compressive strain enhances the maximum power factor (*PF*_max_) of p-type SnSe from 2.3 to 4.3 mW·m^−1^·K^−2^. Specifically, under a −3% compressive strain, the thermoelectric figure of merit (*ZT*) experiences a 50% enhancement, increasing from 0.18 to 0.27. Conversely, for n-type, tensile strain leads to a 26% rise in the *PF*ₘₐₓ, from 53.6 to 67.6 mW·m^−1^·K^−2^. Notably, the 4% tensile strain increased the *ZT* value of n-type SnSe by 123% from 0.66 to 1.47. Importantly, tensile strain effectively reduces lattice thermal conductivity through enhanced phonon scattering, synergistically improving *ZT* with the enhanced power factor. The results show that strain can effectively improve the thermoelectric properties of SnSe, and that n-type SnSe has great potential in thermoelectric materials.

## 1. Introduction

As the energy crisis and environmental issues worsen, finding and developing efficient, clean and sustainable energy conversion and storage technologies has become a major challenge nowadays. Harnessing the ability to transform heat directly into electricity, thermoelectric materials hold great promise in waste heat recovery, thermoelectric power generating and thermoelectric refrigeration, and have attracted much attention and research [1,2,3]. The dimensionless figure of merit, denoted as the figure of merit (*ZT*), is expressed as $ZT=S^{2}\sigma T/\kappa$. This formula is commonly employed to gauge the thermoelectric conversion efficiency of thermoelectric materials. Here, *S* stands for the Seebeck coefficient, *σ* represents the electrical conductivity, *T* is the temperature, and κ indicates the thermal conductivity of the material. Additionally, *PF = S*^2^*σ* is called the power factor [4,5]. The *ZT* value reflects the equilibrium relationship between the *S*, *σ* and *κ* of the material. Consequently, raising *PF* or decreasing *κ* are the primary approaches for improving *ZT* [6,7]. However, the individual parameters are coupled with each other and affect each other, which makes the improvement of thermoelectric properties limited [1,8,9]. Therefore, how to realize the independent or synergistic regulation of the electric and thermal transport properties has become a long-term goal in the current research of thermoelectric materials [10].

As one of the hotspots in the field of thermoelectric materials, chalcogenides have shown great potential in thermoelectric properties due to their unique physicochemical properties [11]. For example, SnTe [12], SnS [13], SnSe [14], GeTe [15] and other materials have made remarkable progress. Among them, SnSe thermoelectric material is an abundant, low-price, and non-toxic semiconductor material with excellent thermoelectric properties, which has attracted wide attention [4]. SnSe has a variety of crystal structures, and the current research mainly focuses on the high-temperature Cmcm phase and Pnma phase. In addition, SnSe with a cubic structure also shows broad application prospects [16]. The Pnma phase SnSe undergoes a phase transition at high temperature, and finally exists stably in the Cmcm phase at high temperature. This structure makes its carrier mobility higher, and the phonon scattering is enhanced, so that the Cmcm phase has excellent thermoelectric properties at high temperature. Guo et al. [17] systematically revealed the uniaxial strain effect of high-temperature Cmcm phase n-type SnSe, and found that the application of uniaxial strain can change the band gap of the material, thus affecting the electrical transport properties of the material. At the same time, strain can also affect the thermal conductivity and further improve the thermoelectric properties of Cmcm phase SnSe. However, for practical waste heat recovery, thermoelectric materials need to be able to operate efficiently in the low-temperature or medium-temperature range (300–750 K) [18]. SnSe materials are stable in the Pnma phase at near-room temperature and medium-high temperatures, but their thermoelectric performance is poor, which limits the application of SnSe materials in a wide temperature range. At present, various methods are being adopted to optimize the performance of SnSe thermoelectric materials. Among these, doping and alloying are important means to improve the thermoelectric properties of SnSe. Band structure and carrier concentration of materials can be regulated by introducing an appropriate amount of impurity atoms or forming alloys with other elements, so as to improve the thermoelectric properties. DUONG et al. achieved a *ZT* of 2.2 (b-axis) at 773 K using Bi-doped n-type SnSe single crystals [19]; Luo et al. obtained a *ZT* value of 1.14 by alloying orthogonal SnSe with cubic AgSbSe_2_ at 723 K [20]. In addition to the high-temperature Cmcm phase studies mentioned earlier, strain engineering has been successfully applied to various thermoelectric materials [21,22,23,24,25], which can affect the thermoelectric properties while maintaining the crystal structure integrity of the material. Because different materials have different bond lengths and crystal structures, the effect of strain regulation in different chalcogenides is not exactly the same. For example, previous studies have shown that compressive strain can effectively improve the thermoelectric properties of p-type and n-type Y_2_Te_3_ [23] and Er_2_Te_3_ [24], and tensile strain can improve the thermoelectric properties of single-layer ZrSe_2_ [26]. For single-layer SnS [27], both types of strain can optimize its performance. However, from these studies, strain can regulate the thermoelectric properties of materials by changing the band structure of materials, such as promoting band convergence and changing band degeneracy. Yan et al. [28] proved that the crystal structure and electrical transport properties of SnSe can be regulated by high-pressure-induced compressive strain. All of these show that strain engineering can be used as an effective method to improve its thermoelectric performance. Based on the above research background, this paper focuses on the effect of uniaxial strain regulation on the thermoelectric properties of Pnma phase SnSe at near room temperature, and compares the regulation of strain on its thermoelectric properties at medium and high temperatures. The purpose is to provide a more comprehensive and in-depth theoretical basis for the practical application of Pnma phase SnSe materials in a wide temperature range. It is worth noting that our research is similar to the previously mentioned Cmcm-phase SnSe research, both of which are devoted to exploring the effect of uniaxial strain engineering on the thermoelectric properties of SnSe. The results show that the applied strain can change the crystal structure of SnSe, whether it is Cmcm phase or Pnma phase, and then affect the key physical processes such as energy band structure, carrier mobility and phonon scattering, and finally achieve the purpose of regulating thermoelectric properties. However, due to the significant difference in the crystal structure of SnSe between the Cmcm phase and the Pnma phase, the response to strain may be different. In addition, the research by Guo et al. [17] mainly focused on the effect of strain on n-type Cmcm phase SnSe at high temperature, and its research conditions and objects are quite different from Pnma phase SnSe. Therefore, the regulation mechanism and effect on the thermoelectric properties of Pnma phase structure cannot be simply determined based on the research results of Cmcm phase SnSe, which also highlights the necessity and importance of in-depth exploration of Pnma phase SnSe in this study.

This study employs Boltzmann transport theory and first-principles calculations are used to methodically examine how strain affects the thermoelectric characteristics of SnSe. Initially, the electronic properties of layered SnSe materials subjected to both tensile and compressive strains are examined. Subsequently, strain’s effect on the key thermoelectric parameters of SnSe materials is analyzed. Ultimately, the power factor (*PF*) of strained SnSe materials and their *ZT* value are determined. The findings demonstrate that applying strain can greatly improve the thermoelectric characteristics of SnSe. At 300 K, the *ZT* values for p-type and n-type SnSe significantly increase to 0.27 and 1.47, respectively.

## 2. Calculation Method

In this paper, to observe how strain affects the SnSe thermoelectric material, uniaxial strain is added to SnSe, mainly on the y-axis (b-axis) of the chosen crystal. The influence of strain is mainly achieved through altering the lattice constant of the material. However, excessive strain may lead to deformation of the material. Therefore, under the premise of ensuring the stability of the material, this paper mainly studies the application of −4% to +4% strain to SnSe material (‘−’ means applying compressive strain to the material, ‘+’ means applying tensile strain to the material), and optimizes the structure to achieve the most stable structure of the material.

All first-principles calculations in this study are based on the Density Functional Theory (DFT) Vienna Ab initio Simulation Package (VASP) implementation [29,30], combined with semi-classical Boltzmann theory to analyze the thermoelectric properties of SnSe materials. The electron-ion interactions are described using the PAW-PBE pseudopotential (Sn_d 06Sep2000) with a 5s^2^5p^2^ valence configuration (ZVAL = 14.000) under the generalized gradient approximation (GGA) with the Perdew–Burke–Ernzerhof (PBE) exchange-correlation functional [31,32]. After the cutoff energy test, the plane-wave cutoff energy is fixed at 500 eV [33], with a 12 × 23 × 8 k-point grid used for Brillouin zone sampling (Figure S1). During the relaxation process, all atoms are permitted to fully relax to their equilibrium positions. For the electrical transport properties, the BoltzTraP2 software package based on the Boltzmann equation is used to calculate the transport parameters [34]. In the Boltzmann transport equation (BTE) calculation, an 11 × 24 × 9 k-point grid that has undergone convergence testing is used (Figure S2). To determine the relaxation time (*τ*) and carrier mobility (*μ*), the deformation potential (DP) theory is utilized [35]. Additionally, the thermal conductivity (*κ_ι_*) of the lattice is computed using the Slack model [36], which then allows for the determination of the *ZT* in SnSe material.

## 3. Results and Analysis

### 3.1. Structure and Electronic Properties of SnSe

**Figure 1:**
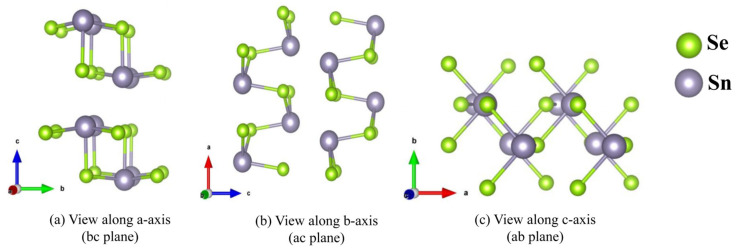
Crystal structure of SnSe.

At atmospheric pressure, the crystal structure of SnSe is an orthorhombic crystal system, belonging to the space group Pnma [37]. The primitive unit cell comprises eight atoms, containing four Sn and four Se. Its lattice constants are a = 4.28 Å, b = 4.30 Å, and c = 11.99 Å. In order to better study the regulation of uniform strain application on b-axis thermoelectric properties, we choose 2 × 1 × 1 supercell to calculate its thermoelectric parameters. Prior to applying strain, the initial structure was optimized using the VASP 5.4.4 software to attain the most stable configuration. The optimized lattice constants are a = 9.18 Å, b = 4.19 Å, and c = 11.75 Å. Figure 1 shows the crystal structure along the a-, b-, and c-axis (bc, ac, and ab planes).

To verify that the SnSe structure is stable and ensure that there are no phase transitions under applied strain, we conducted a 20 ps Ab Initio Molecular Dynamics (AIMD) simulation at 300 K using a 2 × 3 × 1 supercell. The simulation results are given in Figure S3. The total energy of every system showed only minor fluctuations during the course of the simulation. Moreover, we use the 2 × 2 × 1 supercell to compute the phonon spectra under diverse strain conditions, as depicted in Figure S4. Clearly, the phonon spectra for each case under study have no imaginary frequencies. Furthermore, no bond breaking or structural reconstruction was seen throughout the simulations. All of these results indicate the stability of the SnSe structure under strain.

On the basis of stability, we calculated the electronic properties of SnSe. The band structure and density of states are very important for thermoelectric performance analysis [38]. The results indicate that applying strain can effectively regulate the band gap of SnSe and control its electrical transport properties by adjusting the density of states near the Fermi level. For the calculation of bandgap values, within the framework of density functional theory (DFT), the Hartree–Fock exchange Hybrid Functional HSE06 [39] significantly improves the accuracy of bandgap predictions by introducing short-range Hartree-Fock (HF) exchange, enabling more accurate descriptions of electronic band structures and transport properties. However, its computational cost is approximately one order of magnitude higher than that of the PBE functional. In contrast, while the PBE functional underestimates the bandgap due to the incomplete elimination of electron self-interactions, it serves as a classical functional that provides consistent and reliable relative trend analysis. It has been widely applied in theoretical studies of strain effects in SnSe and other thermoelectric materials, particularly in investigating relative trend changes. Therefore, to balance accuracy and computational cost, the PBE functional is a suitable choice in this context.

Regarding the calculated bandgap values, when only the PBE functional is used for calculations, the obtained bandgap values show good consistency with some other theoretical calculation results [37,40]. However, when we further introduce the DFT-D3 [41] correction into the calculations, the calculated bandgap value is 0.55 eV (Figure S5). Compared to the experimentally measured bandgap value of 0.86 eV by Zhao et al. [40], this computational result shows a larger deviation. In contrast, the bandgap value obtained without introducing the DFT-D3 correction is 0.61 eV, which deviates less from the experimental value. This phenomenon fully reveals the complex relationship between van der Waals interactions and electronic structure. The DFT-D3 correction method currently employed may not accurately capture the influence of these interactions on the bandgap. Notably, the trends in bandgap changes observed in PBE calculations and DFT-D3 calculations are highly consistent. Therefore, all results presented in subsequent studies are based on PBE functional calculations.

Figure S6a–i show the band structure calculated at strain between −4% and 4%. The findings demonstrate that the SnSe material always exhibits an indirect band gap. Conduction band minimum (CBM) is located in Γ-Y direction, whereas valence band maximum (VBM) is situated along Γ-X direction, and the predicted band gap is 0.61 eV, which is less than the 0.86 eV experimental value that Zhao et al. reported [40]. This is because the DFT-PBE method used in this paper underestimates the band gap [42], but has no impact on comparing band structure under various strains. Meanwhile, strain can effectively modulate the bandgap of SnSe material. With the gradual increase in the applied tensile strain, the bandgap increases accordingly, while the compressive strain makes the bandgap decrease accordingly. For example, relative to the unstretched state, the tensile strain (4%) increased the gap by about 36%, while the compressive deformation (4%) reduced the gap by about 59%, which was mainly due to the changes in the valence and conduction bands (Table S2, Figure S6).

**Figure 2:**
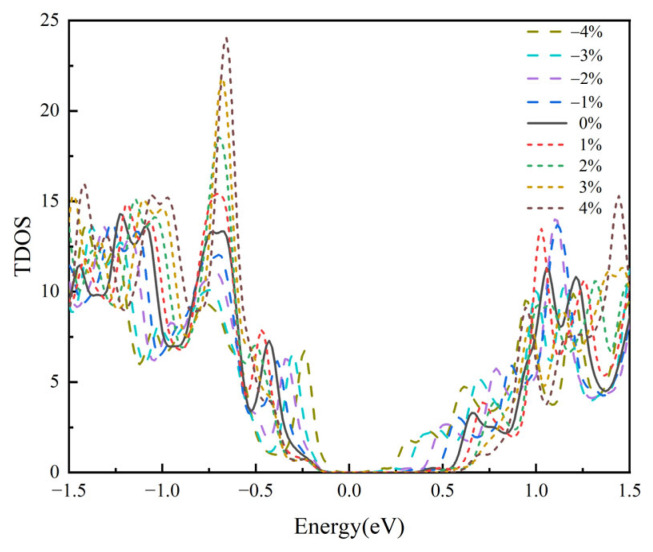
The total density of states (TDOS) distribution of SnSe under strain modulation.

The total density of states (TDOS) diagram for SnSe material is shown in Figure 2. Some researchers have proposed that the characteristics of the density of states (DOS) near the Fermi level can reveal changes in the Seebeck coefficient (*S*) and the electrical conductivity (*σ*) of materials [43,44]. When there are significant fluctuations in the DOS on both sides of the Fermi level, it facilitates the transport of charge carriers through the material. Furthermore, when the DOS peaks are closer to the Fermi level, the density of available electronic states near the Fermi energy is higher. This higher DOS can lead to an increased probability of electron transitions, which in turn can enhance the charge-carrier mobility and thus contribute to higher *σ*. By comparing the valence band and conduction band near the Fermi level in the TDOS diagram, it is found that VBM and CBM are close to the Fermi level under compressive strain. On the contrary, under tensile strain, VBM and CBM will be far away from the Fermi level. Additionally, applying strain causes the slope of the DOS near the Fermi level to increase, which may lead to a larger *S* [45]. The findings from the energy band analysis are in agreement with the changes in the density of states, so they can further reflect the *S* and *σ* changes in materials under strain regulation.

### 3.2. Electrical Transport Properties

The electrical transport properties play a crucial role in determining the performance of thermoelectric materials. The power factor (*PF*) serves as a vital indicator for gauging the electrical transmission features of these materials. It is primarily dictated by two parameters, namely the Seebeck coefficient (*S*) and electrical conductivity (*σ*). To examine how strain affects the electrical transmission characteristics of SnSe, semi-classical Boltzmann theory and BoltzTraP software are used to calculate the electrical transport characteristics of SnSe under various stresses [34]. The focus is on exploring the effect of strain on the *S* and *σ*, uncovering its influence on the *PF* of SnSe materials.

**Figure 3:**
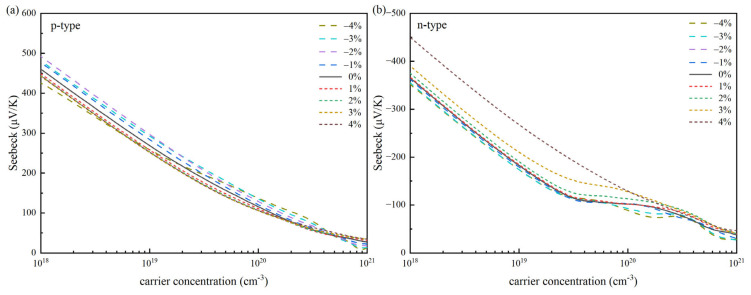
Seebeck coefficient (*S*) as a function of carrier concentration for SnSe at 300 K under strain modulation: (**a**) p-type SnSe and (**b**) n-type SnSe.

Firstly, our investigation focused on how strain influences the *S* in SnSe. As shown in Figure 3, it illustrates the changes in *S* for both p-type and n-type SnSe in relation to carrier concentration under diverse strain circumstances at 300 K. The results indicate that the strain efficiently modulates the *S*, with p-type and n-type SnSe showing similar trends. Under different strains, *S* is approximately linear and diminishes as carrier concentration rises. At a particular carrier concentration, the absolute Seebeck coefficient of p-type SnSe is always higher than that of n-type SnSe without applying strain. For instance, at a carrier concentration of 10^18^ cm^−3^, p-type and n-type SnSe have Seebeck coefficients of 460 μV·K^−1^ and 364 μV·K^−1^, respectively, which may be due to the fact that the valence band near the Fermi level in the band structure is smoother and the DOS of the corresponding valence band is steeper. As illustrated in Figure 3a, for p-type SnSe, compressive strain is more effective than tensile strain. Applying compressive strain enhances the *S*. Conversely, as shown in Figure 3b, the introduction of compressive strain leads to a reduction in the absolute Seebeck coefficient for n-type SnSe, whereas tensile strain raises it. When the tensile strain is 4%, the effect is most noticeable.

The *S* closely relies on the electronic structure of a material in the vicinity of the Fermi level. Therefore, in order to explain how strain influences the change in the *S*, we investigated the inherent relationship between *S* and electronic structure. Based on the energy band theory, the calculation formula of the Seebeck coefficient is expressed as [46]

(1) $$S_{p}=\frac{k_{B}}{e}\left[\ln\left(\frac{N_{V}}{n_{h}}\right)+2.5-r\right]$$

(2) $$S_{n}=-\frac{k_{B}}{e}\left[\ln\left(\frac{N_{C}}{n_{e}}\right)+2.5-r\right]$$

Among them, *S*_p_ and *S*_n_ represent the Seebeck coefficients for p-type and n-type semiconductor materials, respectively. *e* is the electron charge, *k_B_* refers to the Boltzmann constant, *N*_V_ and *N*_C_ are the effective density of states of the valence band and the conduction band, respectively, *r* is the scattering factor, *n_h_* and *n_e_* are the hole and electron carrier concentrations, respectively. Based on Formulas (1) and (2), we can see that the *S* of the material is affected by many physical parameters such as effective DOS, carrier concentration, and scattering coefficient. Among them, *S* has a direct proportionality to the effective DOS. On the contrary, it exhibits an inverse relationship with carrier concentration. It can be found that enhancing the effective DOS is a useful strategy for increasing the *S*. By using strain correction DOS, it is possible to accordingly alter the *S*. At the same time, the DOS analysis of Figure 2 shows that applying compressive strain enhances the effective DOS within the valence band near the Fermi level. This boost directly contributes to an improvement in the Seebeck coefficient for p-type SnSe. Tensile strain lowers the effective DOS, leading to a diminished *S* in p-type SnSe. Similarly, the effective DOS in the conduction band is also affected by strain. Our analysis indicates that tensile strain causes effective DOS to increase, whereas compressive strain causes it to decrease, a change that is consistent with the trend of increasing and decreasing *S* for n-type SnSe. Further analysis shows that the band degeneracy has an important influence on the *S* of SnSe thermoelectric materials. The analysis of the band structure shows that under the action of compressive strain, the originally dispersed band near the VBM gradually approaches and degenerates; under tensile strain conditions, the energy band near the CBM exhibits similar degenerate behavior. This makes the material have more electronic states to occupy when the carrier concentration remains unchanged, which enhances the ability of the material to generate electromotive force under the temperature gradient. At the same time, the increase in the number of energy valleys can increase the effective DOS. When the tensile strain is applied, the number of energy valleys in the conduction band increases, which leads to a significant increase in the *S*. In addition, strain regulation can also affect the relative position and interaction between energy bands. It is found that strain can adjust the energy difference and coupling strength between different energy bands, thereby optimizing the synergistic effect of multiple energy bands, promoting carrier transport and increasing the *S*. Meanwhile, the displacement of the valence and conduction bands relative to the Fermi level induced by strain also reflects the effect of strain on the *S* of SnSe, which can be attributed to changes in carrier concentration. These results indicate that the strain changes the band structure, thus adjusting the concentration of electrons and holes.

Next, we investigated how strain affects the electrical conductivity (*σ*) of SnSe. *σ* is another important parameter affecting thermoelectric properties. Relaxation time *τ* is crucial for assessing material conductivity, and DP theory enables its efficient and rapid computation. Therefore, when calculating the conductivity, we use the DP theory combined with the effective mass approximation to calculate the relaxation time *τ* of the SnSe material, and obtain the relaxation time *τ* and its parameters, m* [47,48]. Table 1 and Table S3 present the calculated findings. Among them, the calculation results of the effective mass m* without strain are consistent with the calculation results of the reference [49] (the effective masses of electrons and holes are 0.11 *m_e_* and 0.31 *m_e_*, respectively, where *m_e_* is the static mass of the electron), which proves the reliability of the results. Since the slope of CBM near the Fermi level is smaller than that of VBM (see Figure 2), this indicates that m* of the electron is lower than that of the hole. According to Formula (1), electron relaxation time exceeds that of holes, which is consistent with our calculations. Due to variations in the DP constant and effective mass of electrons and holes at various strains, their mobility is also different. For example, at −4% strain, the hole mobility is 149.98 cm^2^·V^−1^·s^−1^, while the electron mobility reaches 2473.06 cm^2^·V^−1^·s^−1^, which is 16 times of the hole mobility. In addition, the relaxation time of electrons and holes also shows great differences. For instance, n-type semiconductor carriers have an electron relaxation time of 262 fs without strain, which is about 10 times that of p-type semiconductor hole relaxation time of 26.81 fs, indicating that n-type SnSe may have better electron transport capacity. It is important to highlight that the DP theory primarily centers on the scattering of phonons and electrons and does not consider optical phonon and impurity scattering. This might lead to an overestimation of the conductivity and relaxation time at low and medium temperatures. [50,51,52]. However, the effect of strain on the conductivity trend was not affected in this study [24].

**Figure 4:**
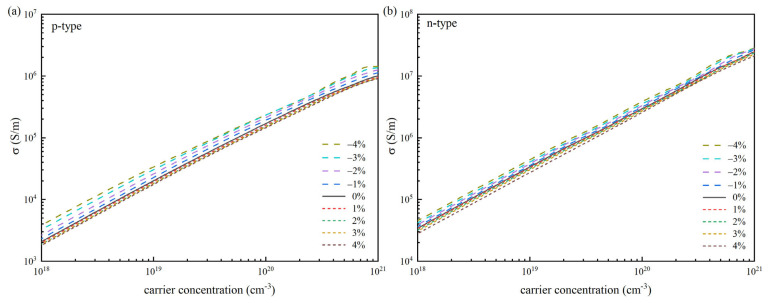
Conductivity *σ* as a function of carrier concentration for SnSe at 300 K under strain modulation: (**a**) p-type SnSe and (**b**) n-type SnSe.

Figure 4 depicts the connection between the carrier concentration and the computed *σ* of p-type and n-type SnSe under strain regulation at 300 K. The results show that for all strain-controlled types, the *σ* increases approximately linearly as carrier concentration increases, with the range of 10^18^ and 10^21^ cm^−3^. By comparing and analyzing the effects of different strains on the *σ*, we found that n-type SnSe boasts a conductivity roughly tenfold higher than its p-type counterpart, primarily due to the disparity in electron and hole relaxation times. For the two types of SnSe, the effect of strain on their conductivity is the same, which increases with the increase in compressive strain and decreases with the increase in tensile strain.

Figure 3 and Figure 4 demonstrate that in thermoelectric materials, the *S* tends to drop as carrier concentration rises, whereas conductivity shows an upward trend with increasing concentration. From the perspective of the carrier concentration region, it shows a strong coupling behavior in the middle and low carrier concentration regions. No matter what strain is applied, the *S* in this region will decrease significantly with the increase in carrier concentration, while the *σ* will increase significantly with the increase in carrier concentration. In the case of low carrier concentration, the relationship between the *S* and the strain can be explained by the Mott relationship [1,53]. The *S* is positively correlated with the effective mass m* and negatively correlated with the carrier concentration. The increase in the carrier concentration will make the Fermi level move to the inside of the band, which will lead to a decrease in the absolute value of *S*. For *σ*, according to the formula *σ = neμ* [1], in this range of carrier concentration, the increase in carrier concentration is the main factor to promote the increase in *σ*. Although the mobility *μ* may be affected by strain, the growth rate of n is much larger than the change caused by *μ*. The band structure change caused by the strain is not enough to destroy the coupling trend determined by the carrier concentration. Therefore, we observe that under different strain conditions, the changes in *S* and *σ* with n are consistent. However, in the region where the carrier concentration exceeds 10^21^ cm^−3^, although the overall trend of *σ* is still consistent, the change in *S* has irregular phenomena, such as abnormal decline rate and slight rebound, which deviates from the monotonic decline trend in the region with medium and low carrier concentration. This may be because when the carrier concentration is high, the position of the Fermi level goes deep into the band and enters the non-parabolic region of the band. At this time, various factors such as non-parabolic performance band effect, multi-energy valley effect, and scattering mechanism change will cause *S* to deviate from the prediction based on the parabolic band hypothesis, thus showing irregular behavior. In addition, they show a diametrically opposite relationship with the DOS near the Fermi surface, which further leads to their different variation with carrier concentration. Based on transport theory, the *S* and *σ* are mutually constrained and change in the opposite direction. For p-type SnSe, the two are decoupled to a certain extent, and the compressive strain increases its *S* and *σ* simultaneously. For n-type SnSe, this law is still valid under the condition of strain regulation, indicating that the strain regulation has not broken their coupling, and the two are still interrelated. Therefore, when assessing electrical transport properties of a material, we cannot make judgments based on a single coefficient.

**Figure 5:**
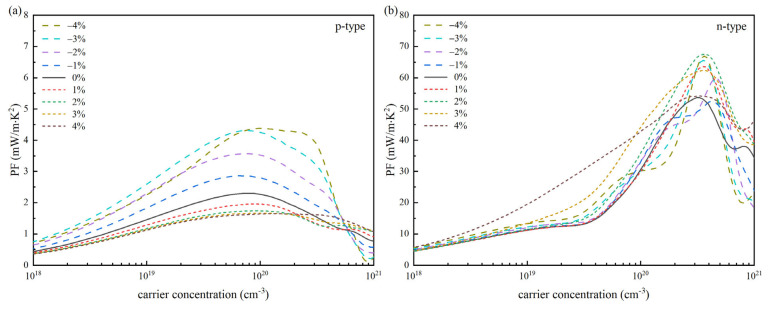
The change in the power factor (*PF*) of 300 K SnSe with carrier concentration under strain regulation: (**a**) p-type SnSe and (**b**) n-type SnSe.

To carry out a more thorough and precise analysis of the electrical transport properties of SnSe, we computed its *PF* at 300 K and obtained a curve that depicts its relationship with carrier concentration (see Figure 5). For p-type SnSe, compressive strain markedly boosts the *PF* across a given carrier concentration range. Under the regulation of strain, the maximum power factor (*PF*_max_) is increased from the original 2.3 mW·m^−1^·K^−2^ to about 4.3 mW·m^−1^·K^−2^, with an increase of about 87%. For n-type SnSe, both compressive and tensile strain can significantly enhance its *PF*. Especially under the tensile strain of 2%, *PF*_max_ increased significantly from 53.6 mW·m^−1^·K^−2^ to about 67.6 mW·m^−1^·K^−2^, which increased by 26%. Therefore, SnSe in its n-type form demonstrates superior electrical transport properties. These results demonstrate that strain regulation can effectively enhance its electrical transport properties.

### 3.3. Thermal Transport Properties

**Figure 6:**
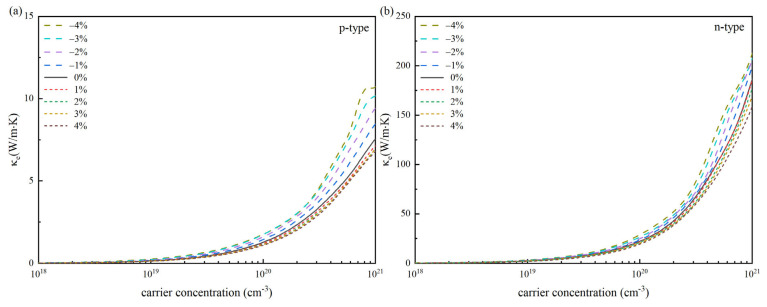
Variation curves of electronic thermal conductivity (*κ_e_*) with carrier concentration of SnSe at 300 K under strain modulation: (**a**) p-type SnSe and (**b**) n-type SnSe.

To more precisely determine the *ZT* of SnSe material, the impact of strain on its heat transport characteristics must be considered, which mainly involves electronic thermal conductivity (*κ_e_*) and lattice thermal conductivity (*κ_ι_*). The correlation between *κ_e_* and *σ* follows the Wiedemann-Franz law [52,54,55], and the expression is: $\kappa_{e}=L\sigma T$, where the Lorentz constant *L* is 2.4 × 10^8^ W·Ω·K^−2^. Figure 6 shows the change in *κ_e_* relative to carrier concentration at 300 K. The findings indicate that as carrier concentration increases, so does the electronic thermal conductivity, which increases rapidly, accelerating significantly above 10^20^ cm^−3^. Therefore, it is expected to obtain the optimal *ZT* value for carrier concentrations not exceeding 10^20^ cm^−3^. Since *κ_e_* is linearly related to *σ*, strain impacts *κ_e_* in a manner analogous to that on *σ* (see Figure 4).

To determine the thermal conductivity *κ_ι_* of SnSe at 300 K, we adopt the Slack model [56,57] and the following is the calculation formula:

(3) $$\kappa_{\iota }\;=\;\frac{0.849\times 3\sqrt[{3}]{4}}{20\pi^{3}\left(1-0.514/\gamma +0.228/\gamma^{2}\right)}\times {\left(\frac{k_{B}\Theta_{\alpha }}{\hbar }\right)}^{2}\frac{k_{B}M_{\alpha }V^{\frac{1}{3}}}{\hbar \gamma^{2}}\times \frac{\Theta_{\alpha }}{T}$$

where *γ* represents the Grüneisen parameter, *Θ_α_* represents the acoustic Debye temperature, *M*_α_ represents the average atomic mass, and *V* represents the volume of the unit cell. Details of calculations based on the slack model are shown in S5 of the Supplemental Materials.

The calculated parameters of SnSe material at 300 K under strains are displayed in Table 2. Strain engineering can enhance the anharmonic effect of materials by weakening the strength of chemical bonds. Grüneisen parameter *γ*, which measures the material’s anharmonic effect strength, is an essential parameter for describing its *κ_ι_*. Stronger anharmonic effects and lower lattice thermal conductivity are associated with greater Grüneisen parameters [58,59,60]. The Debye temperature *Θ_D_* often reflects chemical bond strength and material hardness, correlating with their thermal conductivity. A lower Debye temperature suggests a relatively weak strength of the chemical bond and consequently a relatively low thermal conductivity [61]. It is shown in the table that the transverse sound velocity (v_*t*_), longitudinal sound velocity (v_*ι*_), average sound velocity (v_*m*_) decrease with the increase in tensile strain, which indicates that the propagation speed of phonons in the crystal becomes slower, and the change in sound velocity will indirectly affect the scattering process, which in turn affects the average free path of phonons, and ultimately leads to the decrease in κι. At the same time, when tensile strain increases, the Debye temperature *Θ_D_* falls. This indicates that tensile strain enhances phonon scattering and suppresses phonon thermal transport, thereby reducing the *κ_ι_*. The findings demonstrate that the applied strain makes a substantial impact on the thermal transport characteristics of the SnSe thermoelectric material: tensile strain can effectively decrease the *κ_ι_*, whereas compressive strain will increase it.

The lattice thermal conductivity (*κ_ι_*) calculated by us is 2.96 W·m^−1^·K^−1^, which is different from the value of 2.0 W·m^−1^·K^−1^ obtained by Guo et al. [62], and the calculation result is significantly higher than the experimental data, 0.7 W·m^−1^·K^−1^, along the corresponding direction [40]. This may be due to the obvious anisotropy of phonon transport in SnSe material [63], and the Slack model may overestimate the thermal conductivity. It should be noted that although the Slack model is widely used in thermoelectric materials, it also has some limitations. On the one hand, the Slack model has done a lot of approximations in the derivation process, and the details of the microstructure of the material are not fully considered. Moreover, the fixed or empirical parameters often used cannot reflect the dynamic changes in Debye temperature under different strains, resulting in a certain deviation between the calculation results and the actual situation. On the other hand, for SnSe materials, the Slack model only includes the three-phonon scattering process, and only considering the three-phonon scattering process may overestimate the lattice thermal conductivity (*κ_ι_*) [64]. Moreover, the phonon structure of SnSe is particularly sensitive to temperature [65]. When the temperature increases, the phonon band will be significantly softened, and the Slack model cannot directly describe the influence of the softening of the phonon band on the key physical quantities, such as the phonon frequency. However, in order to balance the computing resources, using the current models and methods, we can still effectively analyze the influence trend of strain on lattice thermal conductivity (*κ_ι_*), so that we can quickly grasp the relationship between strain and thermoelectric properties.

**Figure 7:**
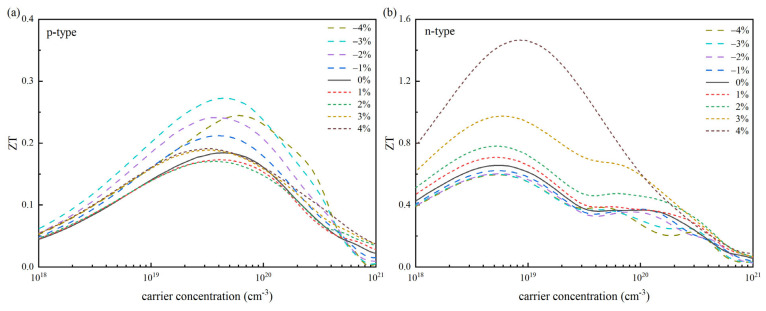
The change in the figure of merit (*ZT*) of SnSe with carrier concentration at 300 K under strain regulation: (**a**) p-type SnSe and (**b**) n-type SnSe.

According to the electrical and thermal transport coefficients, we computed the *ZT* value of SnSe at different strains at 300 K as the carrier concentration varied (see Figure 7). The findings demonstrate that strain engineering effectively enhances the *ZT* value in both n-type and p-type SnSe. In particular, n-type SnSe usually exhibits a higher *ZT*_max_ than p-type SnSe. This is mainly because n-type SnSe has better electrical transport properties than p-type SnSe, that is, higher *PF*_max_. The electronic structures of n-type and p-type SnSe are significantly different, which is an important reason for the difference in thermoelectric properties. For n-type SnSe, the band structure near the bottom of the conduction band is relatively simple and flat, which makes the electron effective mass larger. The larger effective mass of electrons helps to improve the *S*. At the same time, the relatively simple energy band structure makes the electrons less scattered in the transport process, and the high carrier mobility makes the electrons move more quickly in the material, thus ensuring a high *σ* to a certain extent. The band structure near the top of the valence band of p-type SnSe is more complex, and there are multiple energy valleys and band crossings. This complex band structure leads to a relatively small effective mass of holes and uneven distribution, which limits the improvement of the *S*. In addition, the complex band structure also increases the scattering probability of carriers during transport, resulting in a decrease in hole mobility, which in turn affects the *σ* and ultimately limits the thermoelectric properties of p-type SnSe. Moreover, the response of n-type and p-type SnSe to the same strain is also significantly different. When the tensile strain is applied, the band structure change in n-type SnSe is more conducive to the improvement of *PF*. Tensile strain can further optimize the conduction band structure, improve the effective mass of electrons, and reduce electron scattering. At the same time, it can effectively enhance phonon scattering, thereby synergistically improving electrical transport performance, thermal transport performance, and thermoelectric performance. For p-type SnSe, although the compressive strain achieves the synergistic optimization of *S* and *σ*, its overall thermoelectric performance is still inferior to that of n-type SnSe due to the low *PF*.

For p-type SnSe materials, the optimal carrier concentration range for thermoelectric performance is 10^19^ to 10^20^ cm^−3^. Studies have shown that compressive strain can significantly increase its thermoelectric figure of merit (*ZT*). The *ZT*_max_ is increased from 0.18 to about 0.27 by 3% compressive strain, which is increased by 50%. In contrast, the optimal carrier concentration range of n-type SnSe is relatively low (10^18^ to 10^19^ cm^−3^), and the response of its thermoelectric properties to strain shows very different characteristics: compressive strain will lead to a decrease in *ZT* value, while tensile strain can significantly increase *ZT* value. The 4% tensile strain greatly increases the *ZT*_max_ from the initial 0.66 to about 1.47, an increase of 123%. This finding is different from the previous research on the effect of strain on power factor (*PF*), which is mainly due to the synergistic regulation of strain on the thermal and electrical transport properties of materials.

As a material with excellent medium-high temperature thermoelectric properties, we also systematically calculated the thermoelectric properties of SnSe under strain regulation at 500 K and 700 K, including Seebeck coefficient (*S*), electrical conductivity (*σ*), power factor (*PF*), total thermal conductivity (*κ*_tot_), and thermoelectric figure of merit (*ZT*) value (Figures S7–S11). From the results obtained, *S* and *σ* of the material show different characteristics with the change in carrier concentration under different strains, which is due to the influence of bipolar conduction effect. The bipolar effect is particularly significant at high temperature and low carrier concentration [66]. This is because the increase in temperature will promote the intrinsic excitation and produce more electron–hole pairs. The introduction of strain will change the energy band structure of the material, affect the effective mass and mobility of electrons and holes, and then affect the bipolar conduction process. Specifically, when compressive strain is applied, the band gap becomes narrower, which further promotes intrinsic excitation and enhances the bipolar effect. On the contrary, tensile strain broadens the band gap and inhibits the bipolar effect to a certain extent. In our results, the differences in *S* and *σ* curves under different strain conditions can be partially attributed to the regulation of strain on the bipolar effect. For n-type SnSe, the influence of strain on the thermoelectric figure of merit (*ZT*) is consistent under different temperature conditions. For p-type SnSe, in the middle and low temperature range (300 K and 500 K), −3% compressive strain has the best effect on improving the *ZT* value of p-type SnSe. When the temperature rises to 700 K, although the variation trend of power factor (*PF*) and total thermal conductivity (*κ*_tot_) with strain is similar to that at 300 K, the regulation effect of heat transport is more prominent at high temperature. That is to say, in a high-temperature environment, the regulation effect of heat transport is better than that of electrical transport, and the significant reduction in thermal conductivity plays a key role in the improvement of *ZT*. Therefore, at 700 K, although the compressive strain can still greatly improve the *ZT* value of p-type SnSe, the improvement effect of the *ZT* value is the best when 4% tensile strain is applied.

The experimental study of Zhao et al. [67] shows that when the carrier concentration of p-type SnSe is 1.2 × 10^19^ cm^−3^, its *ZT* value at 300 K is about 0.2, which is highly consistent with the p-type data calculated based on the first-principles in this work. The simulation prediction for n-type SnSe shows that the *ZT* value can reach 0.5 at the same carrier concentration, which is significantly higher than the 0.25 reported in the literature. This difference may be attributed to the systematic deviation between the theoretical model and the experimental conditions. This study uses a relatively idealized model. In the modeling process, we ignore many complex factors, such as defects that will inevitably exist in actual materials. At the same time, the DP theory may lead to a higher *ZT* value due to overestimation of relaxation time and conductivity, which in turn leads to a higher calculated *ZT* value. From the actual situation, such a high conductivity presented in the model is extremely difficult to achieve in the experimental operation if relying only on doping. Moreover, the defects generated by the dopants introduced to achieve high concentration doping will inevitably have a negative impact on the thermoelectric properties of the materials [68]. In view of the above situation, in the follow-up research and practice, we can try to adopt a more comprehensive control strategy. On the one hand, the microstructure and electronic state distribution of the material are changed by applying mechanical stress and strain, thus affecting its thermoelectric properties; on the other hand, low-concentration doping combined with strain engineering is used for synergistic regulation. Low-concentration doping can reduce the negative impact of defects on thermoelectric properties to a certain extent. At the same time, combined with strain engineering, it is expected to more accurately optimize the carrier concentration and transport characteristics of materials, thereby improving thermoelectric properties. In addition, the intrinsic carrier concentration of SnSe material is at a low level. In actual experimental operations and specific application scenarios, the carrier concentration is often difficult to achieve the ideal range corresponding to the optimal *ZT* value. However, due to the use of a unified and standardized calculation method for all strain conditions throughout the research process, the trend of strain regulation effect still has reliability and reference value, which can provide a solid theoretical basis for further exploration of thermoelectric performance optimization of SnSe materials.

The results show that the strain has a significant effect on the thermoelectric properties of SnSe in a wide temperature range, and this effect shows different characteristics in p-type and n-type SnSe. At 300 K, the compressive strain can effectively improve the thermoelectric properties of p-type SnSe, while the tensile strain has a more significant effect on the thermoelectric properties of n-type SnSe. This finding provides an important theoretical basis for optimizing the performance of SnSe-based thermoelectric materials through strain engineering. For similar materials, there are differences in the regulation of strain on their thermoelectric properties. For example, Raveena Gupta et al. [27] applied uniaxial tensile and compressive strains to single-layer SnS, and found that the power factor was the largest at 1% tensile strain, and the lattice thermal conductivity was the lowest at−4% strain. At 300 K, both types of strain can optimize the *ZT* value of p-type and n-type SnS. Qin et al. [26] applied tensile strain to single-layer ZrSe_2_, the band degeneracy increased, resulting in an increase in the Seebeck coefficient, while the lattice thermal conductivity decreased, and the thermoelectric properties of p-type and n-type ZrSe_2_ were improved.

## 4. Conclusions

In this study, first-principles calculations and semi-classical Boltzmann transport theory were used to explore the regulation of −4% to 4% strain on the thermoelectric properties of SnSe materials. From our results, strain has a significant effect on the maximum power factor (*PF*_max_) in terms of electrical transport. At different temperatures, for p-type SnSe, compressive strain can significantly increase *PF*_max_. At 300 K, compressive strain increases *PF*_max_ from 2.3 to 4.3 mW·m^−1^·K^−2^, an increase of 87%. And its enhancement effect is better than that of tensile strain, while n-type SnSe has a significant increase in *PF*_max_ under tensile strain, from 53.6 to 67.6 mW·m^−1^·K^−2^, an increase of 26%, indicating that its electrical transport performance is better. At 500 K and 700 K, the *PF*_max_ of the two increases continuously with the increase in temperature. At the same time, *S* and *σ* change with strain and temperature, which jointly affect *PF*. In terms of thermal transport, compressive strain increases the lattice thermal conductivity (*κ_ι_*), while tensile strain decreases it, and this trend is still obvious at different temperatures. Overall, strain has a significant effect on the thermoelectric properties of SnSe materials at different temperatures, with n-type SnSe typically exhibiting a higher *ZT*_max_ than p-type. At 300 K, Compressive strain can increase the *ZT*_max_ of p-type SnSe by 50%, from 0.18 to 0.27. Tensile strain can increase the *ZT*_max_ of n-type SnSe from 0.66 to 1.47, an increase of 123%, showing better thermoelectric properties. At 500 K and 700 K, the *ZT*_max_ advantage of n-type SnSe is further expanded.

Therefore, strain engineering is an effective means to adjust the thermoelectric properties of SnSe, and n-type SnSe has great potential for thermoelectric material applications. Accurately controlling the type and size of strain can optimize its thermoelectric properties and further promote the development and application of thermoelectric devices.

## Figures and Tables

**Table 1:** Effective mass m*, carrier mobility *μ* and relaxation time *τ* of SnSe under strain modulation at 300 K.

Stain (%)	Carrier Type	m* (*m_e_*)	*μ* (cm^2^/Vs)	*τ* (fs)
−4%	Electron	0.211	2473.06	298.18
	hole	0.356	149.98	30.51
−3%	Electron	0.216	2332.36	287.88
	hole	0.363	142.84	29.63
−2%	Electron	0.220	2227.83	280.07
	hole	0.370	136.17	28.79
−1%	Electron	0.224	2129.69	272.60
	hole	0.377	129.93	27.99
0%	Electron	0.230	1993.48	262.00
	hole	0.388	120.92	26.81
1%	Electron	0.231	1971.97	260.30
	hole	0.390	119.40	26.61
2%	Electron	0.232	1950.80	258.62
	hole	0.389	120.16	26.71
3%	Electron	0.232	1950.80	258.62
	hole	0.384	124.10	27.23
4%	Electron	0.232	1950.80	258.62
	hole	0.383	124.92	27.34

**Table 2:** Young’s modulus Y, transverse sound velocity (v*_t_*), longitudinal sound velocity (v*_ι_*), average sound velocity (v*_m_*), Poisson’s ratio (υ), Grüneisen parameter *γ*, Debye temperature *Θ_D_*, acoustic Debye temperature *Θ_α_* and lattice thermal conductivity *κ_ι_* of SnSe material calculated under strain modulation at 300 K.

Stain (%)	Y/Gpa	v_*t*_ (km/s)	v_*ι*_ (km/s)	v_*m*_ (km/s)	υ	*γ*	*Θ_D_* (K)	*Θ_α_* (K)	*κ_l_* (W/mK)
−4	51.21	1.88	3.12	2.08	0.213	1.34	206.46	103.23	3.96
−3	47.73	1.82	3.00	2.01	0.209	1.32	198.74	99.37	3.64
−2	46.53	1.80	2.96	1.99	0.208	1.32	195.70	97.85	3.52
−1	44.18	1.75	2.89	1.94	0.208	1.32	190.03	95.02	3.24
0	41.54	1.70	2.80	1.88	0.207	1.31	183.72	91.86	2.96
1	39.14	1.65	2.72	1.82	0.210	1.32	177.56	88.78	2.63
2	36.57	1.60	2.63	1.76	0.210	1.32	171.34	85.67	2.39
3	33.99	1.53	2.54	1.70	0.214	1.34	164.20	82.10	2.03
4	31.25	1.47	2.45	1.70	0.219	1.36	163.67	81.84	1.96

## Data Availability

The original contributions presented in this study are included in the article/Supplementary Materials. Further inquiries can be directed to the corresponding author(s).

## Supplementary Materials

The following supporting information can be downloaded at https://www.mdpi.com/article/10.3390/ma18174219/s1, Figure S1: Cutoff energy test with a k-point grid of 12 × 23 × 8; Figure S2: Convergence test of k-point grid with a cutoff energy of 500 eV; Figure S3: Evolution of free energy during the 20 ps Ab Initio Molecular Dynamics (AIMD) simulation of the SnSe under strain at 300 K; Figure S4: The phonon spectrum of the SnSe under strain at 0 K; Figure S5: The band structure of SnSe under partial strain modulation obtained by the DFT-D3 method; Figure S6: The band structure of SnSe and the change in VBM and CBM energies as well as band gap with strain under strain modulation; Figure S7: The change of the Seebeck coefficient (*S*) of 500 K and 700 K SnSe with carrier concen-tration under strain regulation; Figure S8: The change of the Conductivity (*σ*) of 500 K and 700 K SnSe with carrier concentration under strain regulation; Figure S9: The change in the power factor (*PF*) of 500 K and 700 K SnSe with carrier concentration under strain regulation; Figure S10: Variation curves of electronic total thermal conductivity (*κ*_tot_) with carrier concentration of SnSe at 500 K and 700 K under strain modulation; Figure S11: The change in the figure of merit (*ZT*) of SnSe with carrier concentration at 500 K and 700 K under strain regulation; Table S1: Comparison of lattice constants corrected by PBE and PBE+DFT-D3; Table S2: Lattice constants, valence band maximum (VBM) and conduction band minimum (CBM) energies, and band gap of SnSe under strain modulation; Table S3: DP constants *E_β_*, elastic constants *C* of SnSe at 300 K under strain modulation. Related to Figure 4.
