# Supplementary material for: First-Principles Study of Strain Engineering Regulation of SnSe Thermoelectric Properties
Source: Materials (Basel). 2025 Sep 8;18(17):4219. doi: 10.3390/ma18174219 (PMC12430063; doi:10.3390/ma18174219)
Supplement: Supplementary file 1 [file materials-18-04219-s001.zip › materials-3833188-supplementary.pdf]

materials

Supplementary Materials

Haoru Zhang [1], Songqing Zhao [1,2,*], Yuhong Xia [1], Xinyue Zhang [1], Lulu Zhou [1] and Zhenqing Yang [1,*]

[1] College of Science, China University of Petroleum, Beijing 102249, China
[2] China University of Petroleum-Beijing at Karamay, Karamay 834000, China
* Correspondence: zsq@cup.edu.cn (S.Z.); yangzhq@cup.edu.cn (Z.Y.)

Academic Editor: Andres Sotelo

Received: 6 August 2025
Revised: 26 August 2025
Accepted: 5 September 2025
Published: 8 September 2025

Citation: Zhang, H.; Zhao, S.; Xia, Y.; Zhang, X.; Zhou, L.; Yang, Z. First-Principles Study of Strain Engineering Regulation of SnSe Thermoelectric Properties. *Materials* **2025**, *18*, 4219. https://doi.org/10.3390/ma18174219

Copyright: © 2025 by the authors. Submitted for possible open access publication under the terms and conditions of the Creative Commons Attribution (CC BY) license (https://creativecommons.org/licenses/by/4.0/).

S1. Convergence test

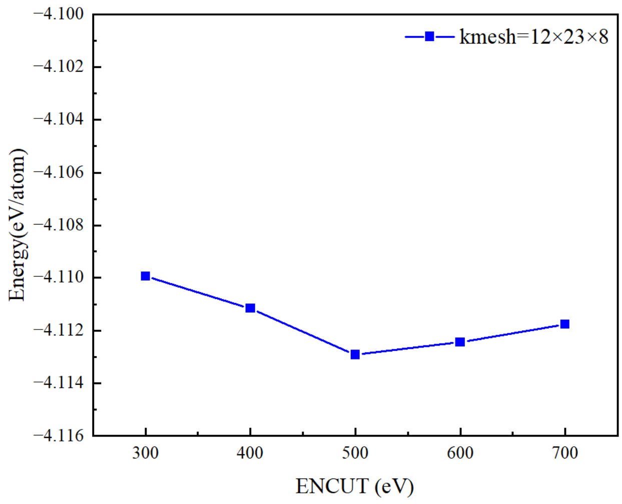

Figure S1. Cutoff energy test with a k-point grid of 12×23×8.

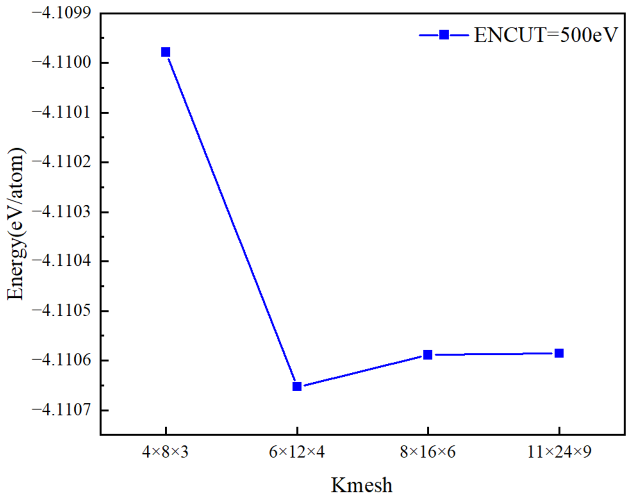

Figure S2. Convergence test of k-point grid with a cutoff energy of 500 eV.

S2. AIMD simulation and the phonon spectra

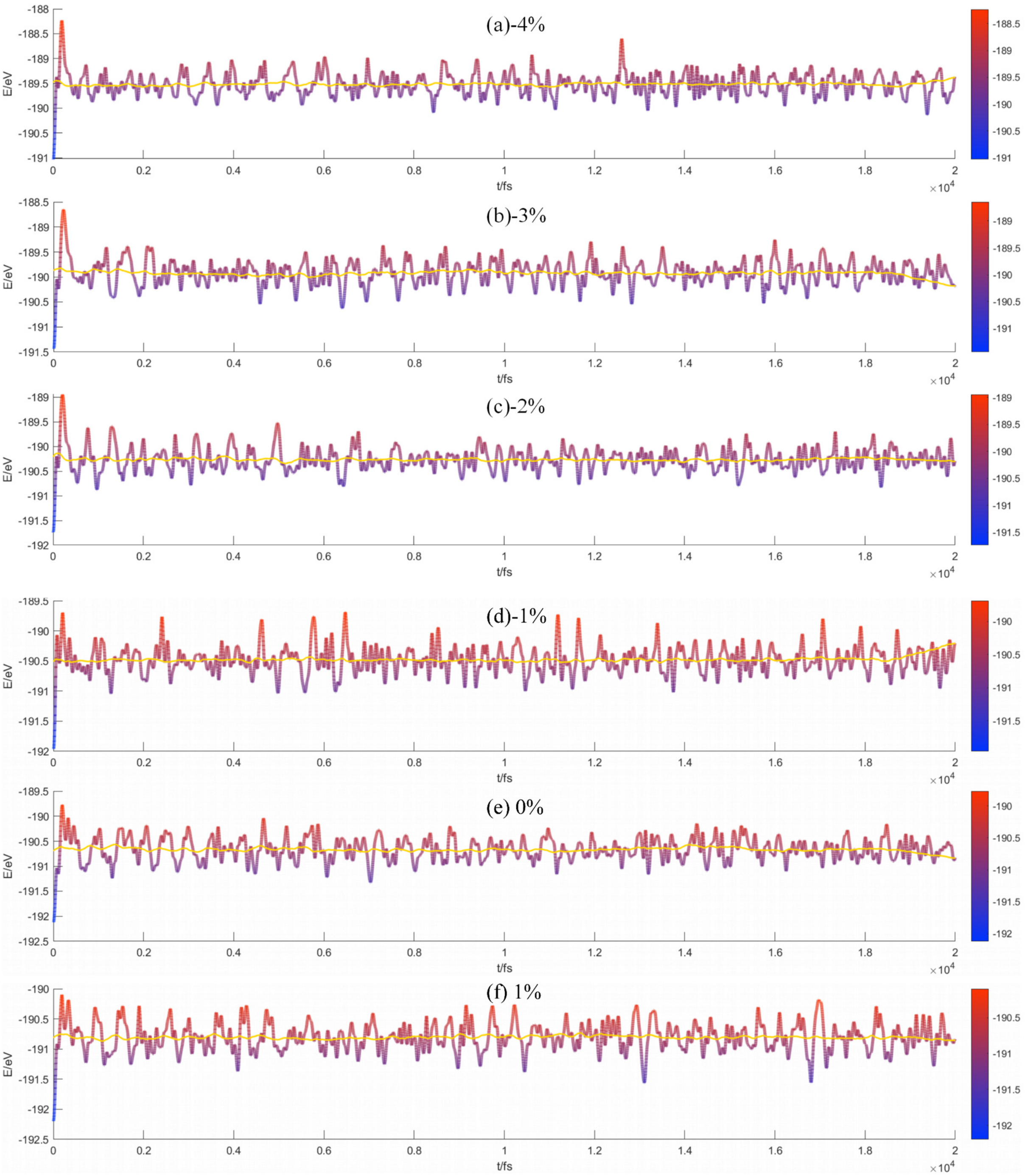
(a)-4%
(b)-3%
(c)-2%
(d)-1%
(e) 0%
(f) 1%
E/eV
t/fs
×10^4

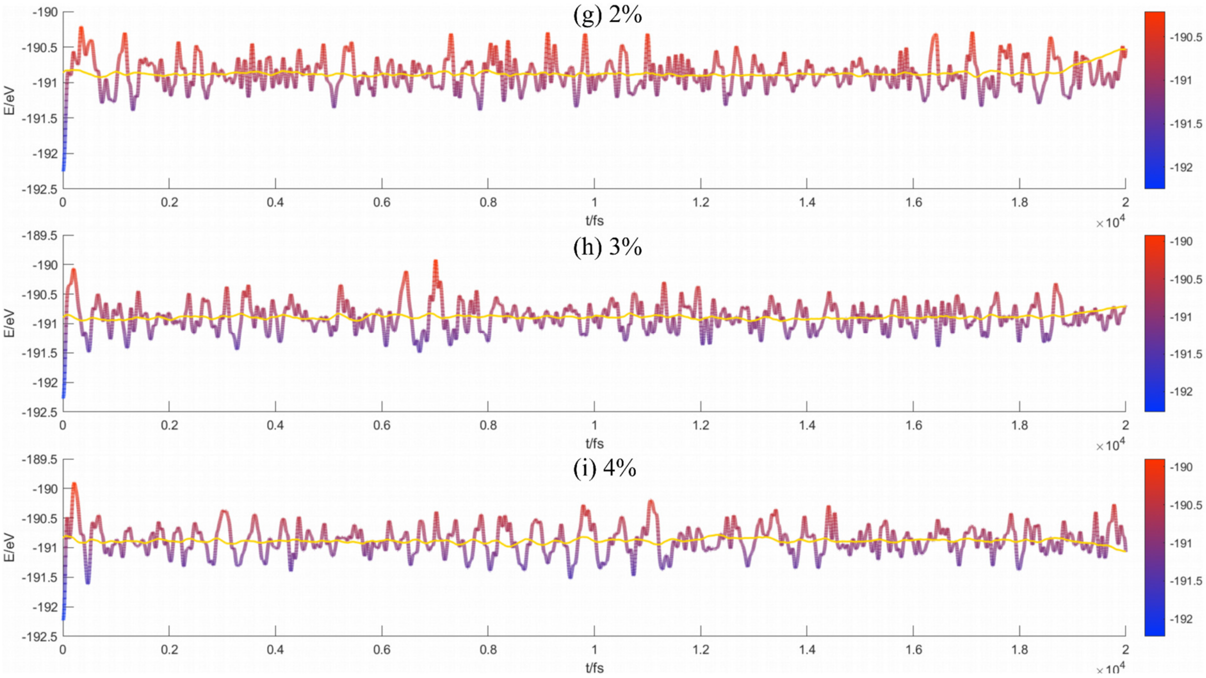

Figure S3. Evolution of free energy during the 20 ps Ab Initio Molecular Dynamics (AIMD) simulation of the SnSe under strain at 300 K.

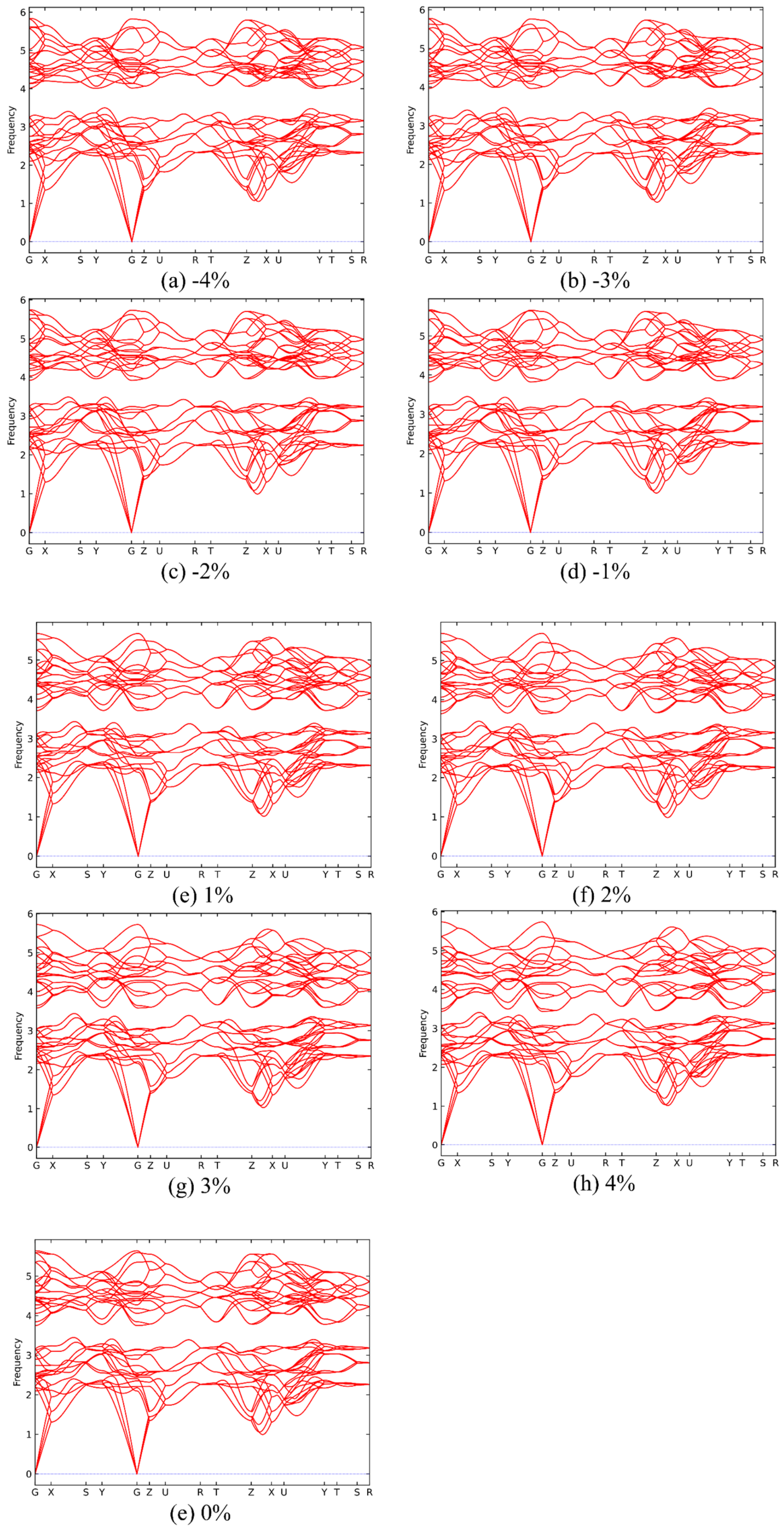

Figure S4. The phonon spectrum of the SnSe under strain at 0 K.

S3. The band structures

For the optimized lattice constant, DFT-D3 correction slightly shrinks the interlayer spacing (c-axis), $\Delta c \approx 0.21$Å, and has an impact of less than 1% on the a-axis and b-axis, as shown in Table S1. At the same time, the band structures calculated by the two methods are highly consistent in shape and band edge position, and the electronic structure trend is consistent (Figure S3).

Table S1. Comparison of lattice constants corrected by PBE and PBE+DFT-D3.

strain (%)	crystal axis	PBE	PBE+DFT-D3	variation value of lattice constant
	a	9.18	9.16	0.02
-0.04	b	4.03	3.99	0.04
	c	11.75	11.54	0.21
	a	9.18	9.16	0.02
0	b	4.19	4.16	0.04
	c	11.75	11.54	0.21
	a	9.18	9.16	0.02
0.04	b	4.36	4.32	0.04
	c	11.75	11.54	0.21

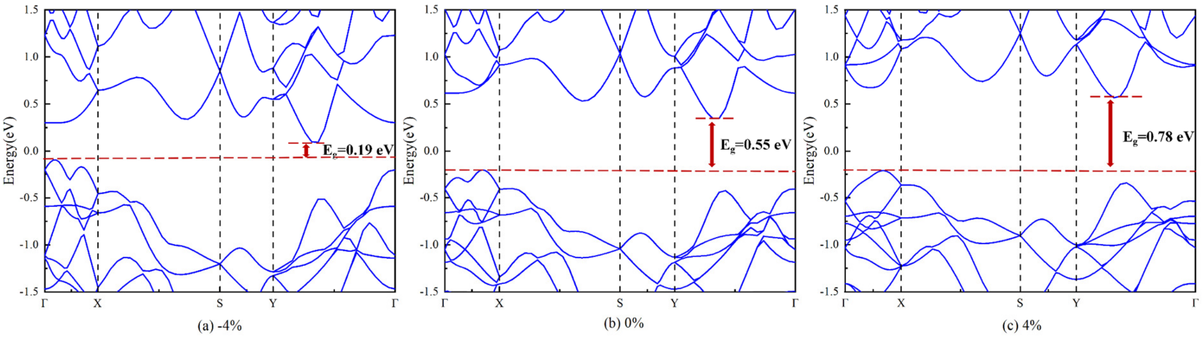

Figure S5. The band structure of SnSe under partial strain modulation obtained by the DFT-D3 method.

Table S2. Lattice constants, valence band maximum (VBM) and conduction band minimum (CBM) energies, and band gap of SnSe under strain modulation.

Strain/%	b/Å	E_{CBM}/eV	E_{VBM}/eV	E_g/eV
-4	4.02	5.65	5.4	0.25
-3	4.06	5.59	5.22	0.37
-2	4.11	5.54	5.06	0.48
-1	4.15	5.48	4.93	0.55
0	4.19	5.41	4.8	0.61
1	4.23	5.34	4.68	0.66
2	4.27	5.29	4.56	0.73
3	4.31	5.23	4.44	0.79
4	4.36	5.16	4.33	0.83

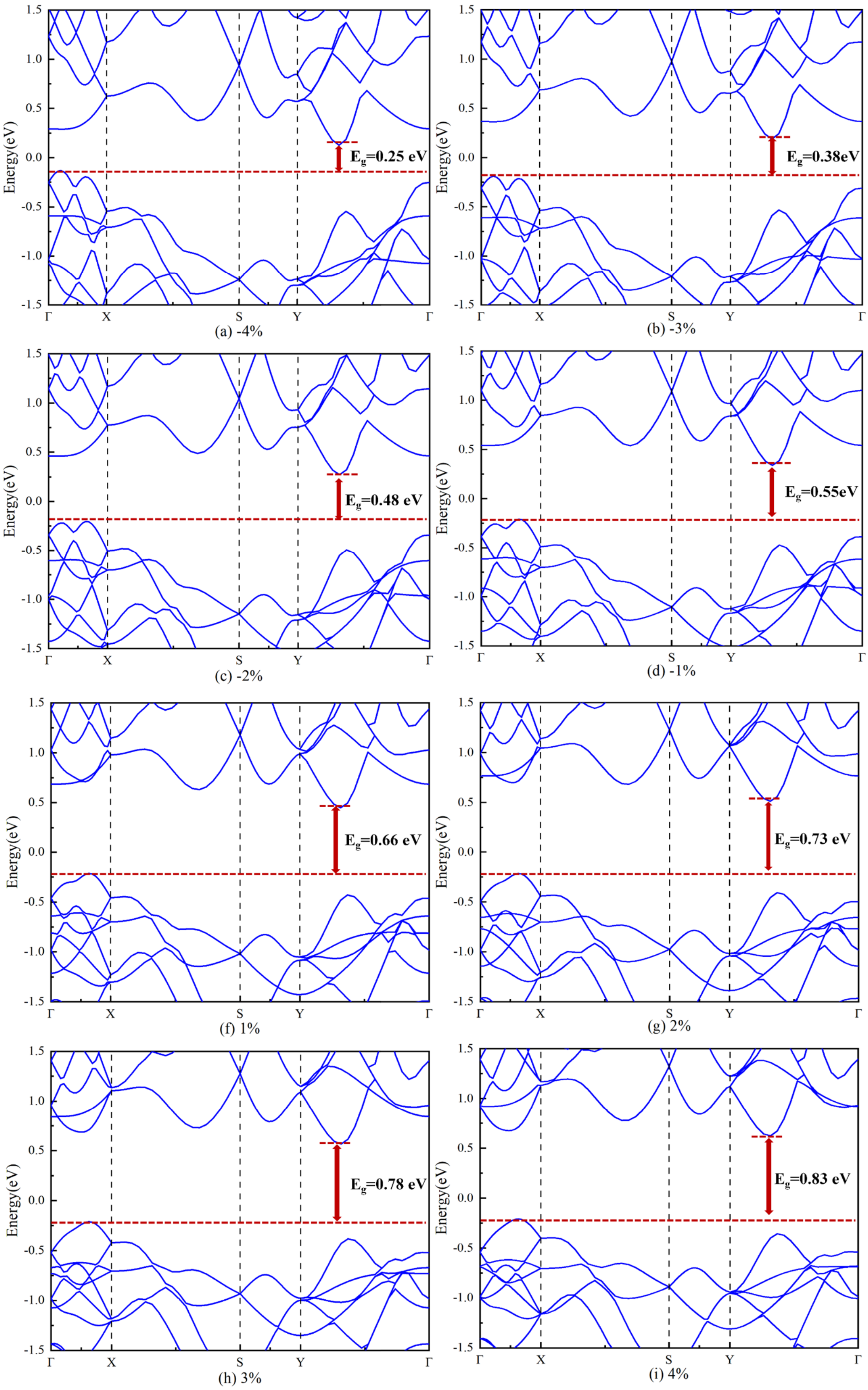

Energy(eV)
Γ X S Y Γ
E$_g$=0.25 eV
(a) -4%
E$_g$=0.38eV
(b) -3%
E$_g$=0.48 eV
(c) -2%
E$_g$=0.55eV
(d) -1%
E$_g$=0.66 eV
(f) 1%
E$_g$=0.73 eV
(g) 2%
E$_g$=0.78 eV
(h) 3%
E$_g$=0.83 eV
(i) 4%

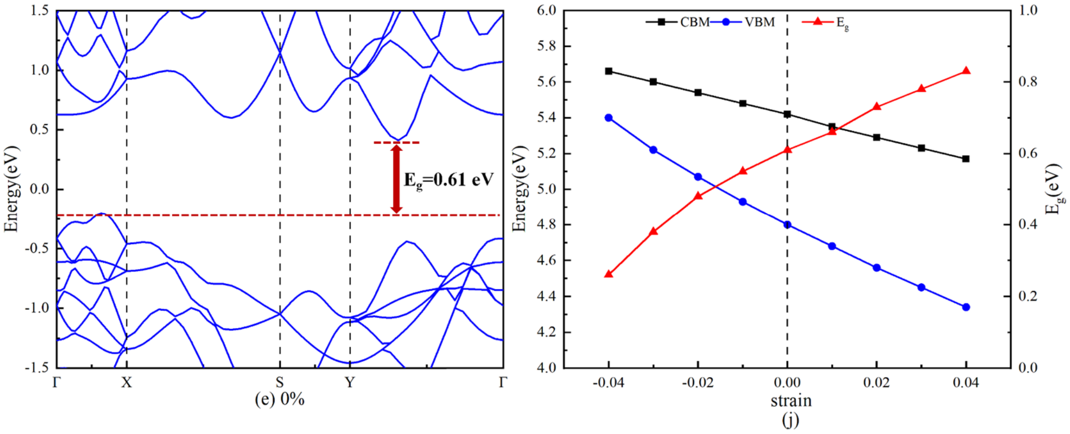

Figure S6. The band structure of SnSe and the change in VBM and CBM energies as well as band gap with strain under strain modulation.

S4. The Parameters Related to the DP Theory

The following formula can be used to calculate how three-dimensional materials' relaxation times vary with temperature:

$$\tau_\beta = \frac{\mu_\beta^{3D} m^*}{e} = \frac{2\sqrt{2\pi}\hbar^4 C_\beta^{3D}}{3(k_B T)^{\frac{3}{2}}(m^*)^{\frac{3}{2}} E_\beta^2} \tag{1}$$

where $\hbar$ denotes the Planck constant, k_B represents the Boltzmann constant, C is the three-dimensional elastic constant, m^* is the effective mass of the charge, T is the temperature, and E_β stands for the deformation potential (DP constant) for the three-dimensional material, indicating its total energy variation under uniaxial strain along the β-axis. m^*, C, and E_β are calculated using the following formulas:

$$m^* = \hbar^2 \cdot \frac{\partial^2 \varepsilon(k)}{\partial k^2} \tag{2}$$

$$C_\beta^{3D} = \frac{1}{V_0}\frac{\partial^2 E}{\partial(\delta l/l_0)^2} \tag{3}$$

$$E_\beta = \frac{\partial E_{edge}}{\partial(\delta l/l_0)} \tag{4}$$

The term E_{edge} represents the movement in the energy band edge, δ_l denotes the variation in the lattice constant relative to its equilibrium value, l_0 signifies the lattice constant along the β direction, and V_0 is the three-dimensional cell volume. According to the calculated m^*, C, and E_β, the relaxation time that depends on temperature can be obtained utilizing the deformation potential (DP) theory. Table S3 gives the DP constant and elastic constant of SnSe under strain modulation at 300 K.

Table S3. DP constants E_β, elastic constants C of SnSe at 300 K under strain modulation. Related to Figure 4.

Carrier type	E_1(eV)	C(*10^{10}J/m $^{-3}$)
Electron	−6.17	3.22
hole	−13.03	3.22

Due to the influence of strain on the energy bands of SnSe material, coupled with the fact that the effective mass (m^*) is intrinsically linked to bands, we have calculated the values of m^*, carrier mobility (μ), and relaxation time (τ) for SnSe under strain modulation at a temperature of 300 K. The carrier mobility is obtained by the variant of formula (1):

$$\mu_\beta^{3D} = \frac{e\tau_\beta}{m^*} \tag{5}$$

S5. Slack model

When calculating the lattice thermal conductivity, we first used density functional theory (DFT) to calculate the elastic modulus matrix of the SnSe system. Subsequently, the bulk modulus (*B*) and shear modulus (*G*) were calculated using the Voigt–Royce–Hill–Gilvarry (VRHG) method. The specific formulas are as follow:

$$B_V = \frac{1}{9}(C_{11} + C_{22} + C_{33}) + \frac{2}{9}(C_{12} + C_{13} + C_{23}) \tag{6}$$

$$G_V = \frac{1}{15}(C_{11} + C_{22} + C_{33}) - \frac{1}{15}(C_{12} + C_{13} + C_{23}) + \frac{1}{5}(C_{44} + C_{55} + C_{66}) \tag{7}$$

$$B_R = [(S_{11} + S_{22} + S_{33}) + 2(S_{12} + S_{13} + S_{23})]^{-1} \tag{8}$$

$$G_R = 15[(S_{11} + S_{22} + S_{33}) - 4(S_{12} + S_{13} + S_{23}) + 3(S_{44} + S_{55} + S_{66})]^{-1} \tag{9}$$

Where S_{ij} represents the inverse matrix of C_{ij}. The *B* and *G* are calculated by the arithmetic mean of B_V、G_V、B_R、G_R. The calculation formulas are

$$B = \frac{(B_V + B_R)}{2} \tag{10}$$

$$G = \frac{(G_V + G_R)}{2} \tag{11}$$

Next, based on *B* and *G* of the material, we computed its Young's modulus (*Y*) and Poisson's ratio (υ). The formulas are as follows:

$$Y = \frac{9BG}{3B + G} \tag{12}$$

$$\upsilon = \frac{3B - 2G}{6B + 2G} \tag{13}$$

On this basis, we further use *Y* and υ to calculate the transverse sound velocity (ν_t), longitudinal sound velocity (ν_l) and average sound velocity (ν_m) of the material according to the following formulas:

$$\nu_t = \sqrt{\frac{Y}{2\rho(1 + \upsilon)}} \tag{14}$$

$$\nu_l = \sqrt{\frac{Y(1 - \upsilon)}{\rho(1 + \upsilon)(1 - 2\upsilon)}} \tag{15}$$

$$\nu_m = \left[\frac{1}{3}\left(\frac{2}{{\nu_t}^3} + \frac{1}{{\nu_l}^3}\right)\right]^{-\frac{1}{3}} \tag{16}$$

Where ρ represents the material's density.

Finally, we calculate the Grüneisen parameter (γ), Debye temperature (Θ_D) and acoustic Debye temperature (Θ_α). The formulas are as follows:

$$\gamma = \frac{9 - 12(v_t/v_l)^2}{2 + 4(v_t/v_l)^2} \tag{17}$$

$$\Theta_D = \frac{h}{k_B}\left(\frac{3n}{4\pi V}\right)^{\frac{1}{3}} v_m \tag{18}$$

$$\Theta_a = \Theta_D n^{-\frac{1}{3}} \tag{19}$$

Where h is Planck's constant and n is the number of atoms in the protocell.

S6. Thermoelectric properties at 500 K and 700 K

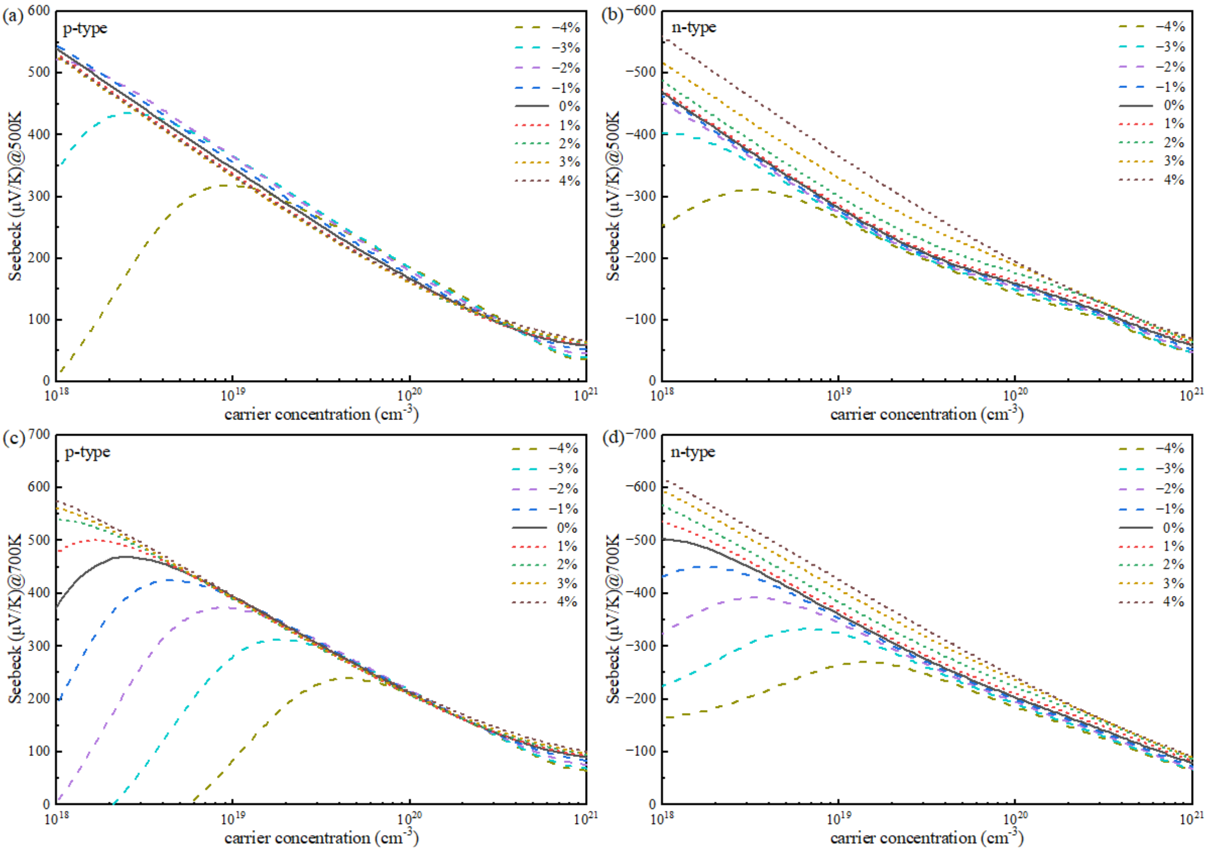

Figure S7. The change of the Seebeck coefficient (S) of 500 K and 700 K SnSe with carrier concentration under strain regulation.

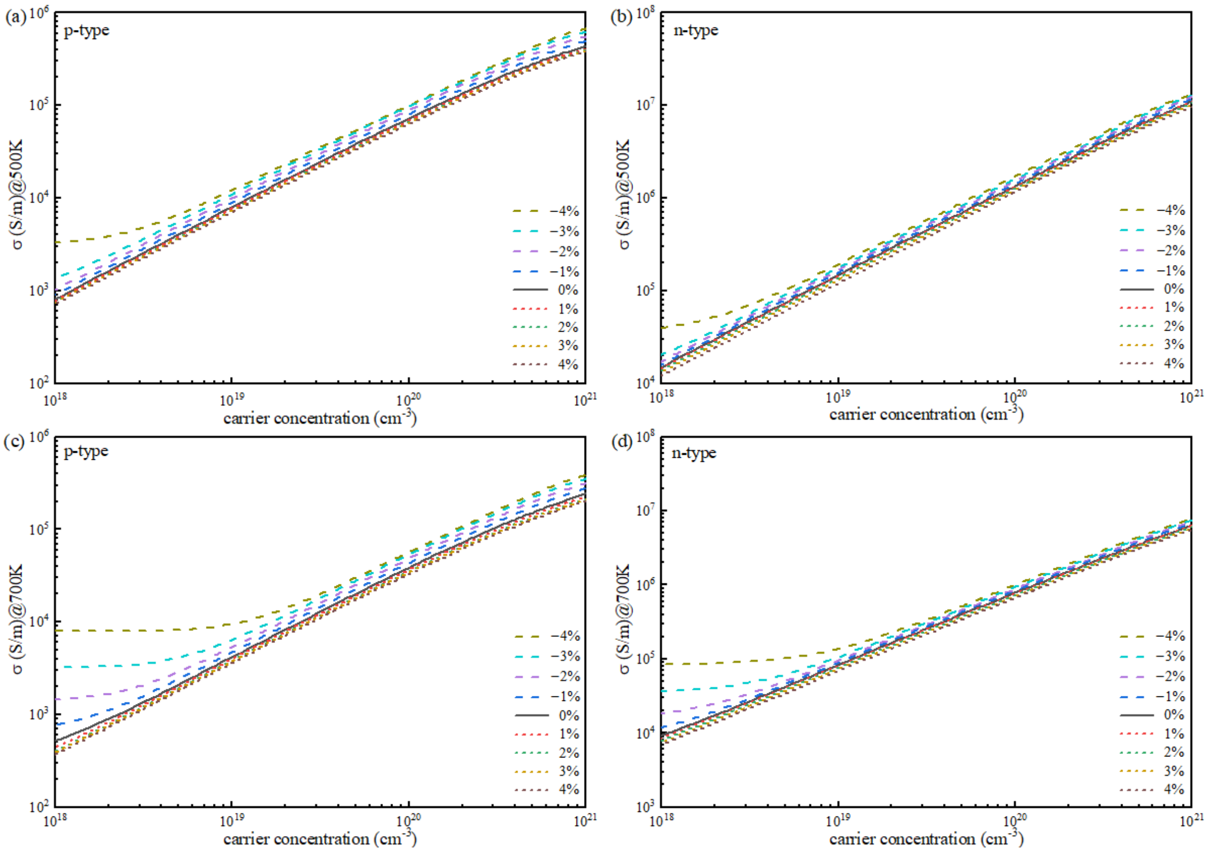

Figure S8. The change of the Conductivity (σ) of 500 K and 700 K SnSe with carrier concentration under strain regulation.

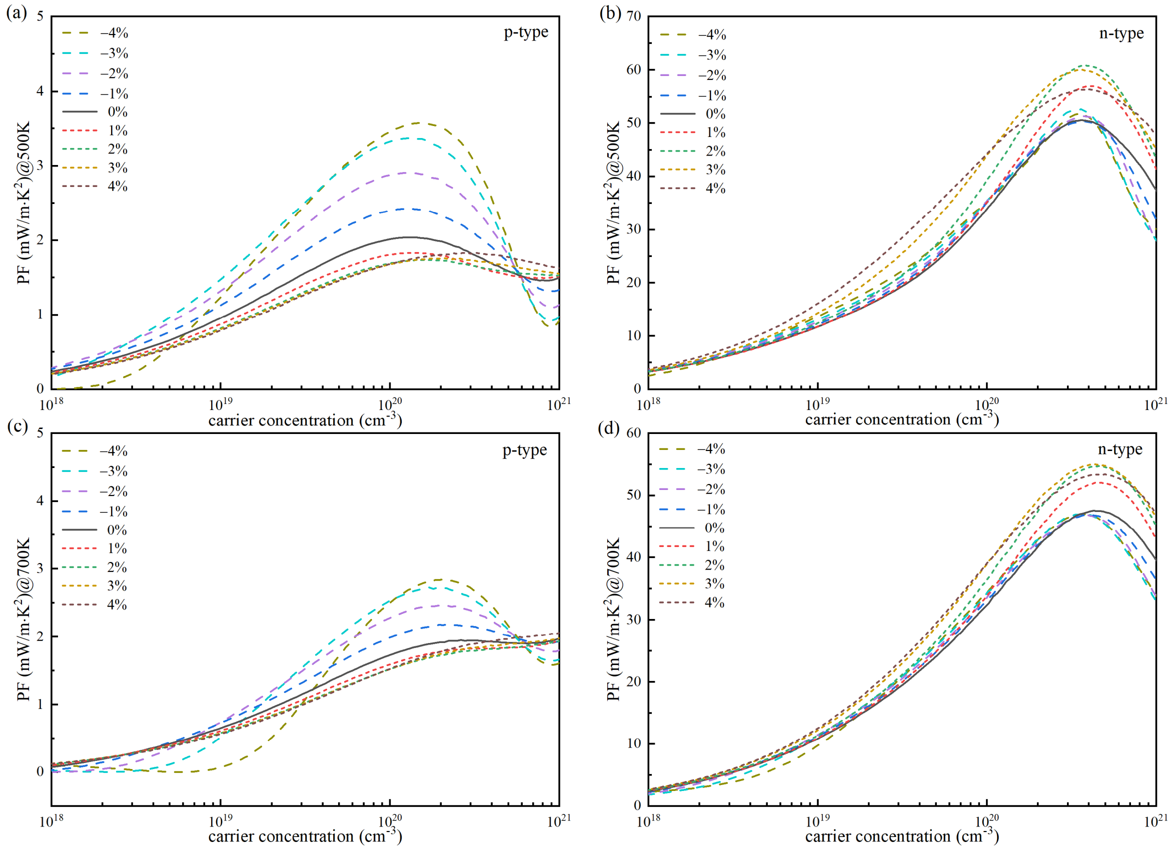

Figure S9. The change in the power factor (*PF*) of 500 K and 700 K SnSe with carrier concentration under strain regulation.

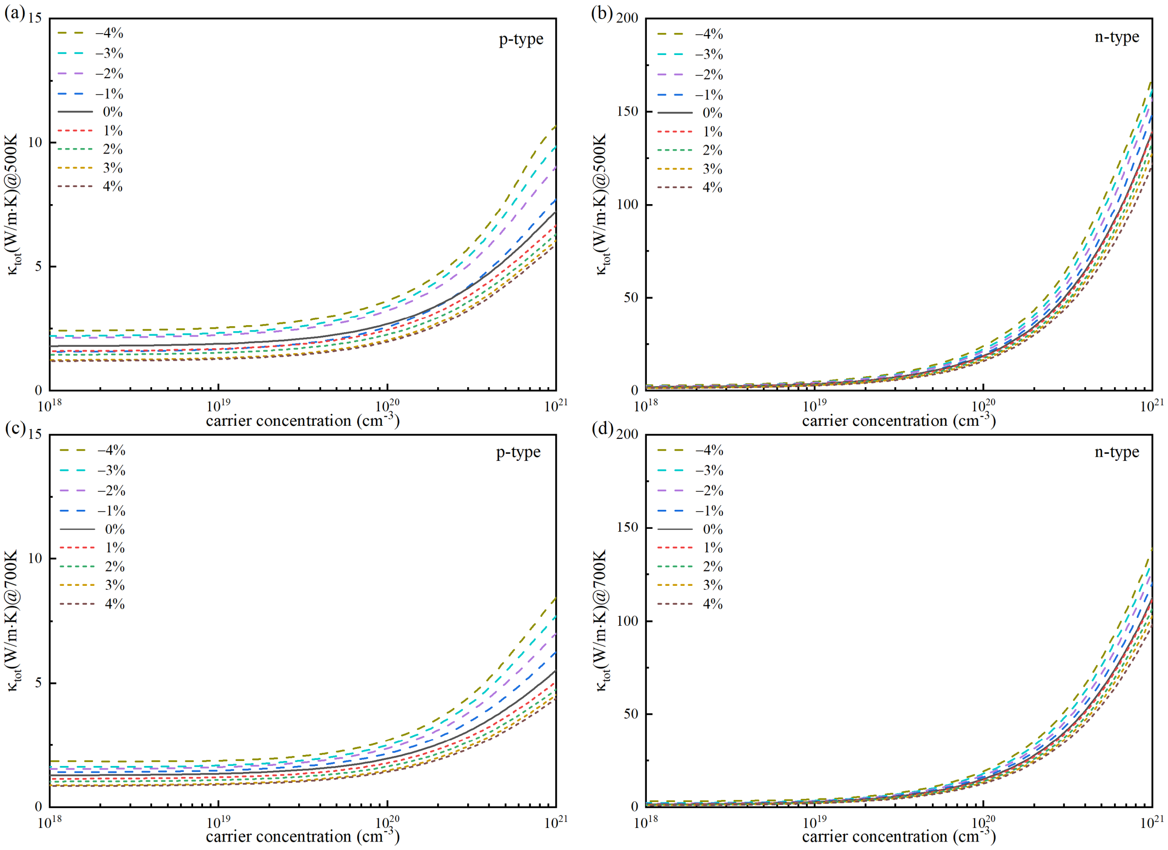

Figure S10. Variation curves of electronic total thermal conductivity (κ_{tot}) with carrier concentration of SnSe at 500 K and 700 K under strain modulation.

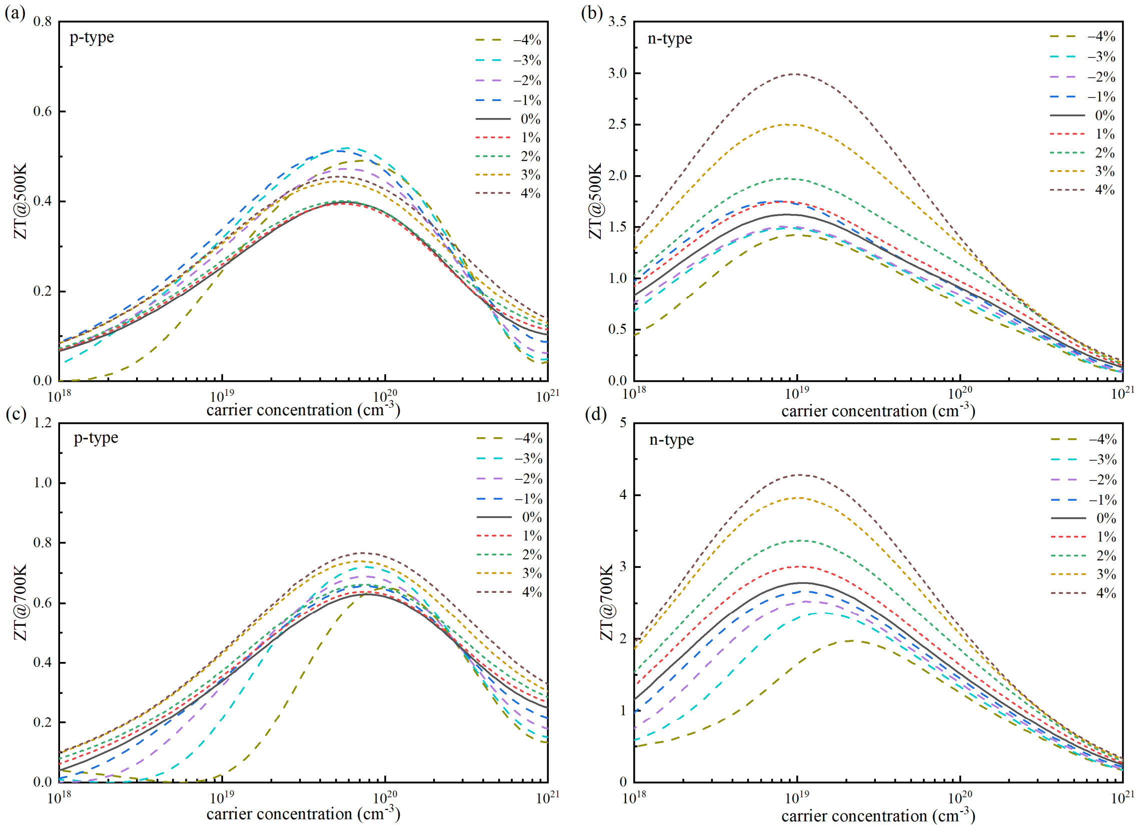

Figure S11. The change in the figure of merit (*ZT*) of SnSe with carrier concentration at 500 K and 700 K under strain regulation.
